# Anisotropic Microparticles with a Controllable Structure via Soap-Free Seeded Emulsion Polymerization

**DOI:** 10.3390/molecules30010166

**Published:** 2025-01-03

**Authors:** Yanping Duan, Xia Zhao, Xiang Nan, Zhifeng Sun, Xiaoyun Lei, Wei Wang, Hong Hao, Jianfang Li

**Affiliations:** 1 Shaanxi Key Laboratory of Catalysis, School of Chemical and Environmental Science, Shaanxi University of Technology, Hanzhong 723001, China; zhaoxia@snut.edu.cn (X.Z.); nanxiang@snut.edu.cn (X.N.); sunzhifeng2019@snut.edu.cn (Z.S.); xiaoyunlei@snut.edu.cn (X.L.); wangwei@snut.edu.cn (W.W.); 2 School of Chemical Engineering, Northwest University, Xi’an 710127, China; 3 Department of Energy and Power Engineering, Shanxi Institute of Energy, Jinzhong 030600, China; lijf@sxie.edu.cn

**Keywords:** anisotropic Janus particles, soap-free emulsions, interfacial polymerization, emulsion stabilization, catalysis

## Abstract

Anisotropic particles have a wide range of applications in materials science such as emulsion stabilization, oil–water separation, and catalysis due to their asymmetric structure and properties. Nevertheless, designing and synthesizing large quantities of anisotropic particles with controlled morphologies continue to present considerable challenges. In this study, we successfully synthesized anisotropic microspheres using a soap-free seed emulsion polymerization method. This approach combines the benefits of seed emulsion polymerization with emulsion interfacial polymerization. By varying the concentrations of dissolved polymeric monomers, 3-methacryloyloxypropyltrimethoxysilane (MPS), and the initiator of potassium persulfate (KPS), different shapes of bowl, cap, and three-sided concave particles were obtained in surfactant-free aqueous solutions, simplifying the post-treatment process. The cap particles are Janus particles with good emulsion stability to toluene/water emulsions over 30 days. The catalytic degradation of 4-nitrophenol (4-NP) was investigated after loading silver nanoparticles on the surface of the particles by in situ deposition. The anisotropic particles obtained in this work have potential applications in emulsion stabilization and catalysis.

## 1. Introduction

In recent years, anisotropic colloidal particles for their asymmetric structure and composition have shown potential applications in the fields of emulsion stabilization [1], oil–water emulsion separation [2], multifunctional coatings [3], drug delivery [4], and catalysis [5,6]. Shapes of anisotropy that have been reported include dumbbells, snowmen, raspberries, discs, rods, porous, single chains, and so on [7,8]. These particles with tunable shapes are prepared by typical surface-selective modifications, microfluidic and seeded emulsion polymerization [9], or some innovative synthetic methods [10].

Seed emulsion polymerization is a classical method that enables the preparation of colloidal particles with uniform shape, narrow particle size distribution, and diverse chemical properties in large quantities [11,12]. In addition to the preparation of snowman or dumbbell-shaped anisotropic colloidal particles using crosslinked [13,14] or hydrophilic seeds [15,16], non-spherical particles such as hemispheres, crescents, caps, etc., prepared using non-crosslinked seeds have attracted attention due to their unique structures [17]. For example, hemispherical particles with an equatorial plane were more effective in reducing the oil–water interfacial area and interfacial tension than the curved surface provided by the dimeric Janus particles, used as a colloidal particle surfactant for Pickering emulsions to stabilize oil-in-water emulsions of hexadecane/water without chemical modification [18]. The crescent-shaped Janus particles use their topology and surface wettability to trap tiny oil droplets and self-assemble in a close-packed pile at the interface of larger oil droplets [19]. The cap-shaped Janus carrier effectively interlocked with micro-capillaries and nanofragments on the leaf surface after loading with insecticide. This interaction creates a ‘hanging cap’ topological effect, which enhances the retention and scour resistance of the carrier on the leaf blade [20].

The team of Wang [21,22] preloaded non-crosslinked polystyrene (PS) seeds into 1-chlorodecane, styrene (St), divinylbenzene (DVB), and 2,2′-Azobis(2-methylpropionitrile) (AIBN) mixed oil droplets, with acrylic acid (AA) solution as the aqueous phase, and prepared oil-in-water emulsions of PS/St/DVB in AA stabilized by surfactant sodium dodecyl sulfate (SDS). Crescent-shaped PSDVB⊃PAA Janus particles with concave hydrophobic side and projecting hydrophilic measurements were prepared after emulsion interfacial polymerization, and large-scale synthesis of Janus particles with a variety of topological features, such as bread, pistachio, crescent, etc., was achieved by adjusting the concentration of the PS polymer and the monomers to modulate. The highly hydrophilic monomer was replaced with micro-hydrophilic 4-vinyl benzyl chloride (VBC), anisotropic benzyl chloride-containing P(St-co-DVB-co-VBC)/PS particles were prepared via emulsion interfacial polymerization and egg-shaped, bowl-shaped, and hemispherical particles could be obtained by adjusting the mass of the VBC monomer [23]. Except for using DVB as the crosslinking agent, PS nanospheres as a substrate, 3-methacryloxypropyltrimethoxysilane (MPS) as a precursor, and the addition of potassium persulphate (KPS) and ammonium hydroxide as an initiator and a catalyst, Janus nanoparticles were obtained by accurately adjusting the rates of the two types of polymerizing [24]. However, these reports, like the classical seed emulsion polymerization to synthesize anisotropic particles, have added surfactants to stabilize the emulsion or to modulate the particles’ asymmetric structure. The addition of surfactants increases the cost of separation and also has the disadvantage of altering the physical properties of the polymer particles [25]. The preparation of Janus particles by soap-free seed emulsion polymerization has also been reported [26,27], but the particle morphology is still mostly classical snowman or dumbbell-shaped. Although monodisperse non-spherical colloidal particles with cavity structure were prepared by simple one-pot soap-free emulsion polymerization of St, MPS, and AA, the mechanism of formation of non-spherical colloidal particles also differed from PS-mediated interfacial polymerization of emulsions [28].

Herein, we describe a new method for preparing non-spherical anisotropic particles such as bowls, caps, and three-sided concave using non-crosslinked PS particles as seeds and MPS as hydrophilic monomer and crosslinking agent by soap-less emulsion polymerization. The method does not add co-solvents and does not require surfactants, simplifying the reaction conditions and post-treatment process. The Si-OH introduced on the particle surface is easier to modify. These particles, whose polymerization was initiated under aqueous conditions with the water-soluble initiator KPS, have a nanometer size, are not easily soluble in organic solvents, and have the potential to be used as emulsifiers and catalyst carriers for Pickering emulsion particles.

To comprehensively understand the mechanisms of non-spherical anisotropic particle formation via surfactant-free seeded emulsion polymerization, the process of cap particle formation was analyzed by online sampling. The effects of seed, solvent environment, initiator, monomer type, and concentration on particle morphology and topography are investigated. The polymerization reaction is rapidly initiated at higher KPS concentrations in the aqueous phase. Particle morphology can be readily manipulated by adjusting the quantities of MPS and St. The prepared samples were used as particulate emulsifiers to stabilize the toluene/water-insoluble system, in which the cap particles had more stable emulsification to toluene and aqueous solutions of different pH. In addition, after loading Ag nanoparticles by in situ reduction, the unique structure of the cap particles accelerated the catalytic degradation of 4-nitrophenol (4-NP).

## 2. Results and Discussion

### 2.1. Preparation and Characterizations of the Anisotropic Microparticles

**Figure 1:**
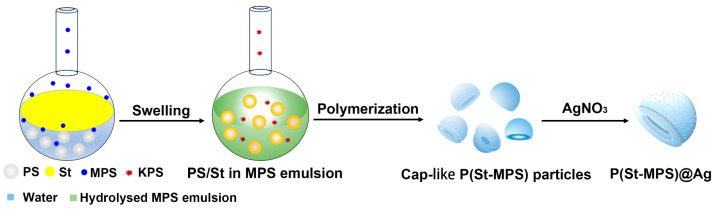
Schematic of the synthesis of P(St-MPS) and P(St-MPS)@Ag microparticles.

Figure 1 illustrates the experimental procedure for synthesizing P(St-MPS)@Ag microparticles. Firstly, non-crosslinked PS seed particles were prepared via soap-free emulsion polymerization. Then, the hydrophobic PS seeds were converted into cap particles with concave surfaces via swelling and interfacial polymerization of a soap-free emulsion using St as the swelling polymerization monomer, MPS as the crosslinking agent and stabilizer, and KPS as the initiator. Finally, P(St-MPS)@Ag composite particles were prepared after the situ reductive loading of Ag nanoparticles. In soap-free emulsion polymerization with non-crosslinked PS particles as seeds, PS seed microspheres were dissolved by St monomer droplets and loosened their polymerization chains. MPS contains both C=C and Si-OCH_3_, and in the presence of water, hydrolyzed and condensed MPS can be used as an emulsifier distributed on the droplets of St containing PS particles to make them stably dispersed in the reaction system. KPS mainly disperses in an aqueous solution, and while increasing the temperature, it initiates polymerization reactions at the interface between St emulsion droplets and water. When the reaction is held for a period, the anisotropic P(St-MPS) polymer particles with controllable structures are obtained via soap-free seeded emulsion polymerization. The morphology of P(St-MPS) particles can be controlled by adjusting the amount and ratio of St and MPS, and cap-like and three-sided concave anisotropic particles were prepared, labeled c-P(St-MPS) and t-P(St-MPS), respectively.

**Figure 2:**
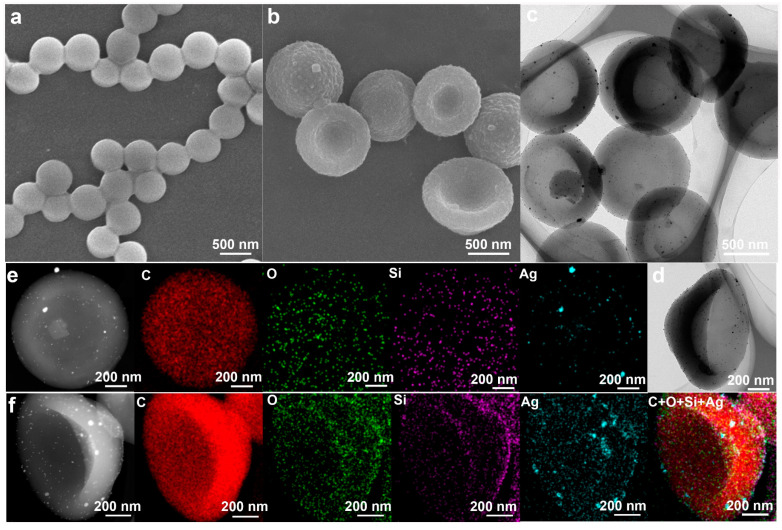
SEM, TEM, and EDX elemental mapping images of samples: (**a**) SEM of PS seeds; (**b**) SEM of P(St-MPS) particles; (**c**,**d**) TEM of P(St-MPS)@Ag particles; (**e**) mapping images of cap’s top facing upwards P(St-MPS)@Ag particles; (**f**) mapping images of side stand P(St-MPS)@Ag particles.

SEM and TEM were employed to investigate the structures of samples. As shown in Figure 2a, the monodispersed PS nanospheres with smooth surfaces were prepared with an average size of 0.58 ± 0.035um, and the high uniformity of PS seeds was further confirmed by DLS, which showed only one intense peak (Figure S1). The cap-shaped P(St-MPS) particles featured rough inner and outer surfaces and were monodisperse, with an average particle size of 0.92 ± 0.074 µm. The rough bumps on the outer side of the cap particle were more pronounced, likely due to the rapid polymerization of MPS initiated from the aqueous phase (Figure 2b). After loading Ag nanoparticles, the morphology of the P(St-MPS)@Ag particles remained unchanged (Figure S2a and Figure 2c). The Ag nanoparticles or clusters varied in size from 1.6 nm to 36.58 nm, with an average size of 8.68 nm, and were unevenly distributed both inside and outside the cap particles (Figure S2b,c). Notably, there were significantly more Ag particles on the outside of the particles and at the brim compared to the inside (Figure 2d).

Elemental Mapping images of P(St-MPS)@Ag particles, with the cap’s top facing upwards, reveal similar distributions of Si and O elements (Figure 2e). Both elements are distributed in circular patterns on the cap’s outer surface. This corresponds to the rough bumps on the outer surface of P(St-MPS) in Figure 2b, which may be related to the uneven distribution of MPS on the PS surface and the different rates of polymerization of MPS and St. The distribution of Ag elements is also similar to that of Si and O elements, except that the content is less, which may be related to the combination of Ag with Si-OH during in situ reduction. The elemental mapping of the side stand of P(St-MPS)@Ag particles (Figure 2f) shows that Si and Ag elements are more distributed at the outer top of the hat particles and the brim. The elemental content of the P(St-MPS)@Ag particles was tested by taking spots at the top of the cap, at the brim, and the depression inside the cap (Figure S2d). It was found that the outer part of the hat particles contained both C, Si, O, and Ag, the brim contained only Si, O, and Ag, while the inner depression of the hat contained C, Si, and O, but no Ag was detected. Consistent with the results of Figure 2b–f, this suggests that the cap particles are Janus particles with an asymmetric distribution of inner and outer compositions.

**Figure 3:**
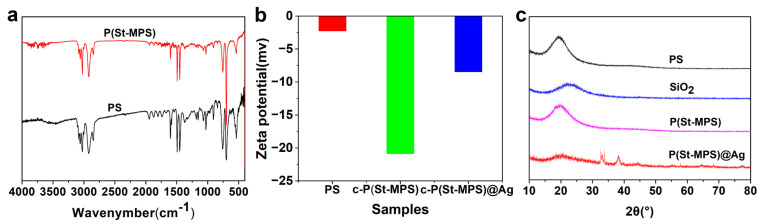
FT-IR (**a**), zeta potential (**b**), and XRD pattern (**c**) of the samples.

FT-IR spectra of PS seed particles and P(St-MPS) particles are shown in Figure 3a. The peak at 3026 cm^−1^ is attributed to the stretching vibrations of C−H in the benzene ring, whereas 2928 cm^−1^ was ascribed to a symmetric and asymmetric 1 stretching vibration of C-H of alkene. The peaks at 1497 cm^−1^ and 1448 cm^−1^ are assigned to the skeleton vibration (C=C) of the benzene ring, whereas the peaks at 696 cm^−1^ and 754 cm^−1^ correspond to the bending vibration of C−H in the benzene ring. These six peaks appeared in the FT-IR spectra of PS nanospheres and P(St-MPS) particles due to the existence of PS. The absorption peak at 1716 cm^−1^ is attributed to the C=O stretching vibration, and the absorption peaks in the 921 cm^−1^ ~1299 cm^−1^ interval are attributed to the stretching vibration absorption peaks of the -C-C-, -C-O-, and -Si-O- bonds, suggesting the introduction of MPS in P(St-MPS) particles.

The surface zeta potential of PS seeds was measured to be −2.24 mV, and the zeta potential of c-P(St-MPS) and t-P(St-MPS) particle surfaces decreased to −20.87 mV and −2.74 mV, respectively. Following the incorporation of Ag nanoparticles, the zeta potential of the P(St-MPS)@Ag surfaces for both morphologies increased to −8.44 mV and −0.253 mV, respectively, as shown in Figure 3b. This indicates that Ag nanoparticles are loaded on P(St-MPS) particles through electrostatic interactions. The bun peak with 2θ of 20° in the XRD pattern of P(St-MPS) and P(St-MPS)@Ag samples is the diffraction peak of polystyrene (Figure 3c), and 38.116°, 44.277°, 64.426°, and 77.47° in P(St-MPS)@Ag correspond to Ag nanoparticles (111), (200), (220), and (311) reflections.

### 2.2. The Formation Mechanism of Anisotropic P(St-MPS) Particles

**Figure 4:**
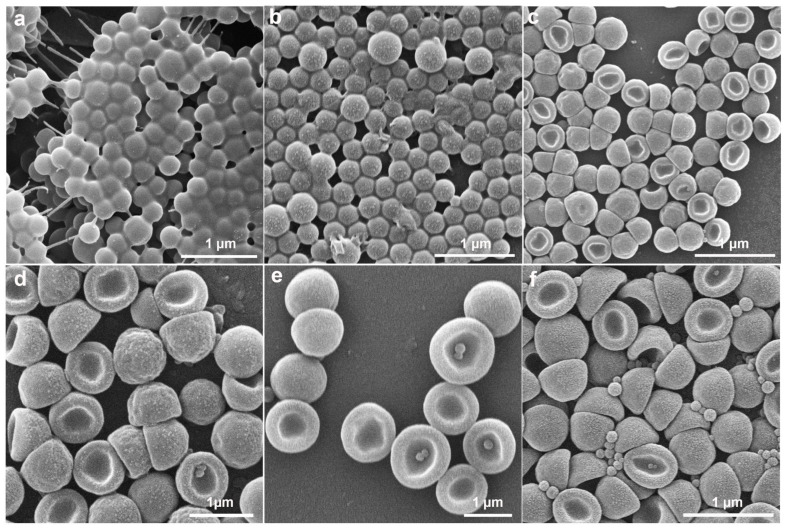
SEM images show the morphology of the samples at different reaction stages: (**a**) dissolution at room temperature for 1 h; (**b**) warming up to 65 °C; (**c**) reaction for 0.5 h; (**d**) reaction for 1 h; (**e**) reaction for 2 h; (**f**) reaction for 8 h.

To understand the formation mechanism of anisotropic P(St-MPS) particles, the evolution of the particle morphology at different stages of the polymerization reaction was tested using SEM, and the change in particle size during the response was statistically determined (Figure 4 and Figure S3). As shown in Figure 4a, the surface of PS seeds was smooth after 1 h of dissolution at room temperature, with a few small punctate particles attached to its surface, and the seed microspheres were partially dissolved and adhered together. The average particle size increased from the original 0.58 ± 0.035 µm to 0.6 ± 0.055 µm (Figure S3). This increase in size suggests that the St monomer’s swelling effect caused the linear PS microspheres to expand. Moreover, MPS hydrolytic condensation forms a reticulation that wraps around the surface of the seed particles from the waterside, and the newly formed organosilicon alcohols condense and grow on the already-formed organosilicon patches [24], creating small particle bumps on the PS surface.

When heated to 65 °C, the particles maintained their spherical shape, but their surface texture turned rough (Figure 4b). Additionally, the average particle size was reduced to 0.57 ± 0.064 µm. This size reduction is likely due to the shrinkage of the PS polymer chains and the migration of PS chains following heating, which led to a decrease in particle size and increased inhomogeneity. Heating up also further promotes the hydrolysis and condensation of MPS making the surface of PS particles rough with small bumps. Monodisperse cap particles were formed when KPS was added to initiate polymerization for 0.5 h. However, the outer surface of the cap particles at this time was not flat and was slightly wrinkled (Figure 4c). As the polymerization reaction proceeded to 1 h, 2 h, and 8 h (Figure 4d–f), the surface of the cap particles gradually became more uniform, yet their overall morphology remained unchanged. Concurrently, the number of small self-polymerized particles that appeared increased. Furthermore, the dimensions of the P(St-MPS) particles stabilized after the polymerization reaction and were allowed to proceed for up to 2 h, as shown in Figure S3.

The analyses suggest that the cap structure of P(St-MPS) particles forms early in polymerization due to the differing polarities and reaction rates of MPS and St. The St monomer dissolves non-crosslinked PS seed particles, causing PS chains to migrate to the oil–water interface. Hydrophilic MPS partially wets and unevenly distributes on St droplet surfaces from the aqueous side and stabilizes the droplets in a soap-free solution. Upon warming and adding water-soluble KPS, polymerization initiates at the aqueous side of the interface. MPS rapidly polymerizes into nuclei and copolymerizes with St. Initially, a high KPS concentration quickly consumes limited MPS, forming cap particles. The PS chains at the interface slow the aqueous phase reaction, leading to uneven contraction of the crosslinked shell and wrinkled cap-like particles. As the reaction proceeds, the remaining MPS and St polymerize, increasing the cap particles’ thickness and height and smoothing their surface.

#### 2.2.1. Effects of Components in the Reaction System on Particle Morphology

The synthesis of polymer particles featuring accessible chemical functionality or specific morphologies typically employs heterogeneous polymerization techniques. The nucleation and growth steps of these reactions are sensitive to solvent polarity and are also related to the solubility and reactivity of the monomer [27]. A series of experiments were conducted to assess the influence of each component within the reaction system on particle morphology.

**Figure 5:**
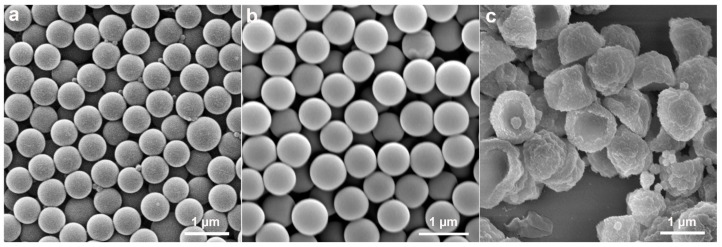
Effect of solvent environment on particle morphology: (**a**) 0.1 wt% Nass solution; (**b**) ethanol–water (1:1 *v*/*v*) mixture solution; (**c**) surfactant-free aqueous solution.

Effects of solvent environment. As shown in Figure 5, spherical P(St-MPS) particles were produced when polymerization occurred in a NaSS solution (0.1 wt%) or an ethanol–water mixture (1:1 *v*/*v*), while seed emulsion polymerization in a surfactant-free aqueous solution yields cap-like particle (Figure 5c). This indicates that surfactant or solvent polarity affects MPS wetting and distribution on PS seed droplet surfaces. Reducing the interfacial tension between MPS and PS seed droplets using a NaSS aqueous solution allows MPS to be uniformly distributed on the surface of seed particles, leading to core–shell particles with slightly rougher surfaces (Figure 5a). When PS seeds, St monomer, and MPS were placed in an ethanol–water mixture (1:1 *v*/*v*), both St and MPS dissolved in the ethanol, co-dissolving the PS seeds. This reduced interfacial tension between seed particles and solvent, resulting in core–shell particles with smooth surfaces (Figure 5b). In surfactant-free aqueous solution, hydrophilic MPS was partially wetted on the surface of hydrophobic PS seed droplets due to the difference in affinity polarity, and non-spherical cap-like particles were prepared from the aqueous phase after initiating rapid polymerization.

**Figure 6:**
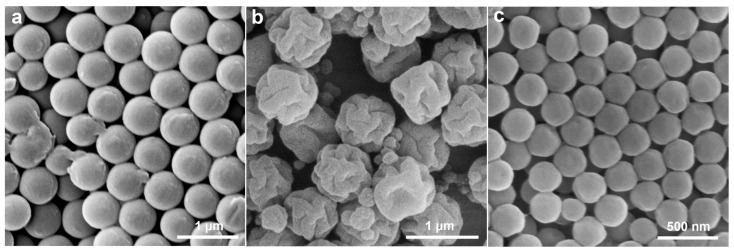
SEM image of the synthesized particles after changing the reaction components: (**a**) add only 2 wt% MPS; (**b**) reduce half the amount of KPS; (**c**) 2 wt% MPS co-polymerized with St to form CPS particles at no PS seeds.

Core–shell PS@PMPS particles were synthesized by adding only 2 wt% MPS as shown in Figure 6a. Each particle has a darker, less conductive circular patch, indicating a different distribution of shell composition. Additionally, the formation of cracks at the junctions with varying shell layer compositions was observed, which may be attributed to the continuous stirring during the reaction process or the weak interfacial adhesion between the shell layer and the cores. In a soap-free solution, hydrophilic MPS does not dissolve PS seeds but partially wets and distributes them on their surface, forming core–shell particles with uneven microphase-separated.

The concentration of the water-soluble initiator KPS affects the rate of free radical polymerization of C=C in MPS and St as well as the rate of diffusion of KPS from the oil–water interface to the seeds droplet. In a typical procedure for the preparation of cap particles in 3.3.1, 45.1 mg of KPS was added to initiate rapid polymerization from the aqueous phase, and since the rate of polymerization in the aqueous phase was much greater than that in the oil phase, cap particles were formed within a short time as observed in the on-line sampling (Figure 4). When the initiator KPS was reduced to half (add 22.6 mg), non-spherical particles with multiple small depressions and folded surfaces were produced, and large and shallow concave surfaces on the particle surface can still be seen (Figure 6b). The reduced KPS dosage slowed the MPS polymerization rate, minimizing the rate difference inside and outside the St droplets. This filled and reduced the concave surfaces inside the emulsion droplets, while the outside formed folds due to the uneven MPS distribution causing crosslinked network contraction. This indicates that the concentration of KPS significantly influences particle morphology. Aligning with the conclusions of the literature [24], subsequent experiments were continued using a higher KPS concentration of 2.5 wt%.

Furthermore, in the absence of PS seeds, only smooth surface crosslinked polystyrene (CPS) particles were produced at the soap-free emulsion polymerization with the same ratio of St and MPS (Figure 6c). The average diameter of CPS particles is smaller than that of PS particles, unlike cap P (St-MPS), which has a particle size much larger than that of PS seed particles. This indicates that the mediating and topological functions of PS seeds in the reaction system are crucial for the formation of concave particles.

**Figure 7:**
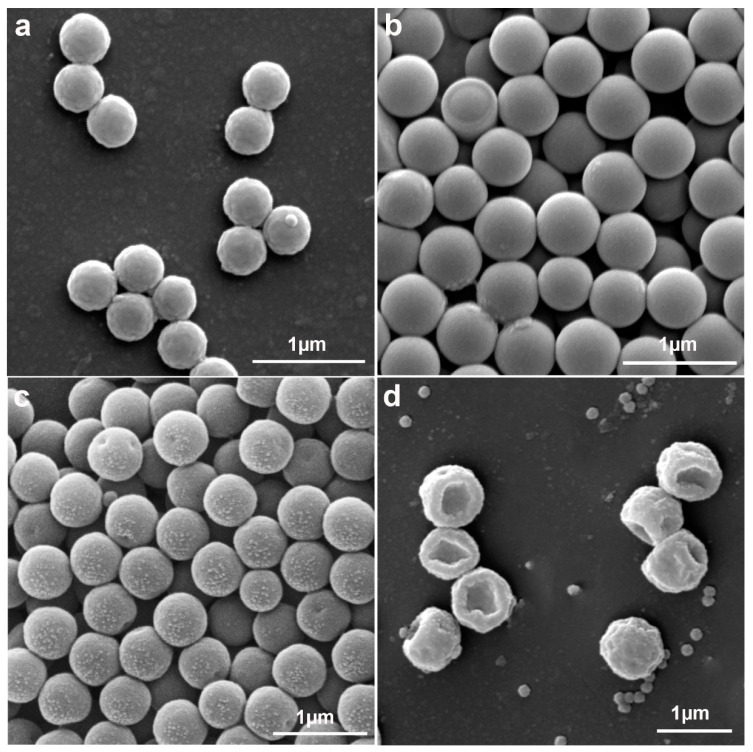
The impact of monomer and crosslinker type on particle morphology: (**a**) MMA+MPS; (**b**) AA+MPS; (**c**) St+DVD; (**d**) St+AA+MPS.

Different monomers have different reactivity and different ability to solubilize seeds. Experiments were conducted using methyl methacrylate (MMA), AA, and a combination of St+AA as polymerization monomers, with a monomer-to-seed mass ratio of 4:1. Additionally, 2% MPS or DVB was added as a crosslinking agent, with the results presented in Figure 5. When MMA served as the monomer and MPS as the crosslinking agent, PS@P(MMA-MPS) core–shell particles with a slightly roughened surface and microphase separation were produced after polymerization, due to MMA’s inability to dissolve PS seeds (Figure 7a). In the soap-free solution, the hydrophilic AA neither dissolved nor completely wet the PS particles, forming non-spherical PS@P(AA-MPS) particles with an inhomogeneous surface shell layer (Figure 7b). As depicted in Figure 7c, when DVB was used as the crosslinking agent, the solution polymerization yielded non-spherical particles featuring two distinct small depressions. The reason for this phenomenon is the uneven distribution of DVB on the PS surface and the different polymerization rates of DVB compared to St, which leads to an uneven crosslinking degree of the shell layer causing concavity. This suggests that the distribution of crosslinking agents on the PS surface and the distribution of crosslinking networks inside and outside the PS also affect the morphology of the non-spherical particles. When polymerized with 95 wt% St + 5 wt% AA as the mixed monomer and MPS as the crosslinking agent, a P(St-MPS-AA) cap particle with a wrinkled surface was obtained (Figure 7d). The above comparisons illustrate that the solubilizing capacity of the monomers used such as St for PS in soap-free solutions and the partial wetting of the hydrophilic MPS with the hydrophobic PS seed surface have a significant effect on the formation of cap-like non-spherical particles. This suggests that the degree of partial wetting between MPS and PS seeds under soap-free conditions with constant MPS addition does not change depending on the type of the other polymeric monomer. In addition, the ability of the St monomer to solubilize the PS seeds is a prerequisite for the formation of non-spherical particles.

#### 2.2.2. Effect of St to PS Seeds Mass Ratio on Particle Morphology

**Figure 8:**
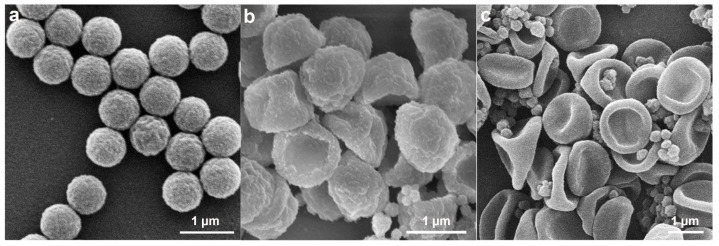
Effect of mass ratio of St to PS seeds on particle morphology: (**a**) mass ratio 2:1; (**b**) mass ratio 4:1; (**c**) mass ratio 8:1.

Effect of St to PS seeds mass ratio on particle morphology. As shown in Figure 8a, at a St-to-PS mass ratio of 2:1, rough surface and microphase-separated core–shell PS@P (St-MPS) particles were obtained. Capped particles (Figure 8b) and three-sided concave particles (Figure 8c) were synthesized when the St-to-PS mass ratios were 4:1 and 8:1, respectively. During the solubilization process, the St monomer diffuses into the PS seed, solubilizing it, while the hydrophilic MPS is partially wetted and distributed across the PS surface. When added in small quantities, St could not fully dissolve the PS seeds, and the St monomer was predominantly distributed within the PS seeds. The crosslinked shell layer generated by the copolymerization of MPS with a small amount of St shrinks, and the PS chain containing St droplets is partially extruded to form a microphase separation, which continues to polymerize to give rough-surfaced core–shell particles. After interfacial polymerization of the soap-free emulsion, the crosslinked shell layer of the concentrated wetted portion of the MPS was uniform and thicker, forming cap particles. When the mass ratio of the St-to-PS seed is too large, on the one hand, PS is completely dissolved in the emulsion droplets of St and the volume expansion is maximum. On the other hand, MPS is diluted by more St and distributed more thinly on the droplet’s surface. The crosslinked shell layer generated after polymerization is too thin to support the particle skeleton, which collapses to form relatively smooth three-sided concave particles, and the excess St with MPS generated many self-polymerizing particles (Figure 8c). This indicates that the amount of St added affects the topology of P(St-MPS) particles. A suitable mass ratio of St to PS seeds ensures adequate dissolution of PS seeds without excessively diluting the concentration of MPS and affecting the hardness and structure of the crosslinked shell layer.

#### 2.2.3. Effects of MPS Concentration on Particle Morphology

**Figure 9:**
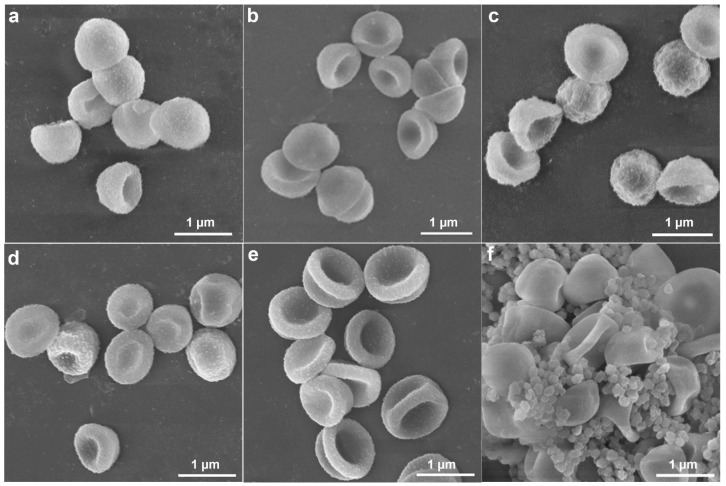
Effect of MPS concentration on the morphology of P(St-MPS) particles: (**a**) 0.5 wt% MPS; (**b**) 1 wt% MPS; (**c**) 2 wt% MPS; (**d**) 3 wt% MPS; (**e**) 6 wt% MPS; (**f**) 10 wt% MPS.

Effect of MPS dosage on particle morphology. Experiments were carried out by adding 0.5 wt%, 1.0 wt%, 2.0 wt%, 3.0 wt%, 6.0 wt%, and 10 wt% of MPS. The results are shown in Figure 9, and with the increase in MPS concentration, bowl-shaped, cap-shaped, cap with a concave surface, and three-sided concave particles were prepared, respectively. The amphiphilic crosslinker, formed through the hydrolysis and polycondensation of MPS, supplies the necessary elastic stress for particle deformation. It also plays a crucial role in maintaining the material’s hardness and surface roughness. With the increase in MPS dosage, hydrolyzed-Si-OH increased, thus regulating the hydrophilicity of St droplets with MPS emulsion, affecting the wetting and distribution of MPS on the surface of PS seeds, with larger openings of the capsid particles and increased roughness of the outer surfaces after polymerization (Figure 9a–c). With the MPS continuing to increase, the crosslinking agent on both the inner and outer surfaces of the cap particles increased, and the elastic contraction of the crosslinked shell layer after polymerization was subjected to an uneven force, and the structure collapsed, resulting in the formation of three-sided concave particles (Figure 9d,e). This three-sided concave particle is more like a deflated hat. In addition to the large depression formed by the original MPS at the interface between the point and face wetting of the PS surface, both sides of the cap also collapse due to the uneven stress on the crosslinked shell layer, forming two relatively small concave surfaces. When too much MPS was added, three-sided concave particles with smooth surfaces were generated, and they stuck together with the small self-polymerized PMPS particles and could not be dispersed (Figure 9f).

Figure S4 shows the EDS elemental analysis of the cap shape and three-sided concave particles prepared at 2 wt% MPS and 6wt% MPS. The elemental content of Si and O on the outer side of the cap particles is higher than that on the inner side of the cap particles, as seen in Figure S4a, indicating that MPS is mainly concentrated on the outer side of the cap particles. Both the ridge portion and the depression of the three-sided concave particles contain Si and O elements, but the Si and O contents of the depression are higher than those of the protruding ridge portion (Figure S4b).

The TG curves of PS, c-P(St-MPS), and t-P(St-MPS) particles were compared (Figure S5), and they had similar trends in weight loss. From the locally enlarged inset a (temperature range 50 °C to 400 °C), it can be seen that the first stage of weight loss of c-P(St-MPS) and t-P(St-MPS) particles occurs around 175 °C, mainly due to the dehydration of their -Si-OH content. The initial stage of weight loss in PS-seeded microspheres occurs at 300 °C due to the decomposition of PS chains. In the temperature range of 400 °C to 475 °C (inset b), the rate of weight loss of PS is faster than that of P(St-MPS), which is because the no-crosslinked PS is more susceptible to carbonation and ablation. The final residue of t-P(St-MPS) of 2.21% was greater than that of c-P(St-MPS) of 1.21%, and with the increase in the concentration of crosslinker MPS, the crosslinking network on the surface of the P(St-MPS) particles increased, and the Si content increased. This suggests that the three-sided concave particles formed by the high MPS content are due to the collapse of the crosslinked shell layer after uneven stress distribution. When the MPS content is large (>3%), soap-free emulsion interfacial polymerization produces three-sided concave particles in all cases.

In summary, the mediating and topological role of hydrophobic PS seeds is the basis for the formation of asymmetric particles by the interfacial polymerization of soap-free emulsions, and the sufficient solubility of monomers to non-crosslinked PS seeds is a prerequisite for the formation of non-spherical particles. Partial wetting and uneven distribution between hydrophilic MPS and hydrophobic PS seeds under soap-free conditions, and the rapid initiation of polymerization from the aqueous side by a high concentration of initiator KPS, are the key control steps for the formation of non-spherical particles. Capped and three-sided concave asymmetric particles can be synthesized by controlling the quality of MPS and St.

### 2.3. Applications of P(St-co-MPS) Microparticles with Surface Functionalities

The emulsion stability of equal masses of PS, spherical CPS particles, and cap-shaped c-P(St-MPS) and three-sided concave t-P(St-MPS) particles were compared in a toluene/water system with a ratio of 2:3 by weight. As shown in Figure S6a, the PS particles stabilized the lowest emulsion layer, primarily located on both sides of the oil–water interface. This is probably due to the negatively charged PS ends and the ability of toluene to dissolve PS. The CPS, c-P(St-MPS), and t-P(St-MPS) crosslinked and copolymerized with MPS were dispersed in the aqueous phase to form oil-in-water(O/W) emulsions, in which the droplets of O/W emulsions stabilized by t-P(St-MPS) were larger particles visible to the naked eye. The EI of the PS-stabilized toluene/water emulsion declines rapidly over time (Figure S6b). This decrease could be attributed to loosening PS chains as they dissolve in toluene. The EI of crosslinked spherical CPS particles stabilizing toluene/water emulsions decreases with time. The c-P(St-MPS) and t-P(St-MPS) had better emulsion stability against the toluene/water system at 30 days, where the EI values of c-(St-MPS) emulsions were higher than those of t-P(St-MPS). Based on the previous characterization analysis, it can be seen that the roughness and hydrophilicity of the inner and outer surfaces of the cap or bowl particles are different, and the asymmetry of the shape of the three-sided concave particles and the difference in the surface properties are less so the emulsion layer formed is lower.

**Figure 10:**
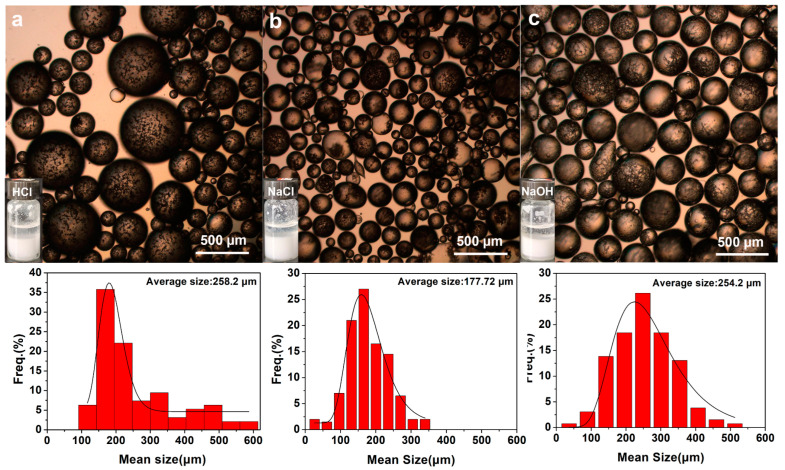
Optical micrographs of cap particles stabilizing toluene/water emulsion droplets at different pH solutions: (**a**) pH = 1 HCl; (**b**) 1 wt% NaCl; (**c**) pH = 13 NaOH.

Emulsion stabilization of anisotropic particles in real environments such as industrial wastewater and high-salt solutions is simulated. Figure 10 and Figure S7 show optical micrographs of emulsion droplet formation when c-P(St-MPS) and t-P(St-MPS) particles were used as particulate emulsifiers to stabilize toluene/HCl (pH = 1), toluene/NaCl solution (0.1 wt%), and toluene/NaOH solution (pH = 13), respectively. As shown in Figure 9c, visible cap particles and three-sided concave particles can form oil-in-water emulsions in excessively acidic, excessively alkaline, or high-salt solutions to toluene. The cap particles stabilized toluene oil emulsion droplets in 0.1 wt% NaCl solution with a minimum mean particle size of 177.72 µm. The toluene emulsion droplets stabilized in HCl and NaOH solutions had larger particle sizes, with average sizes of 258.2 µm and 254.2 µm, respectively. The three-sided concave particles in Figure S7 formed toluene emulsion droplets of larger sizes in HCl, NaCl, and NaOH solutions with average sizes of 269.37 µm, 387.15 µm, and 328.13 µm, respectively. The stabilization of emulsion droplet size by P(St-MPS) particles in different pH solutions may be related to the interaction of -Si-OH contained on its surface with H^+^ or OH^-^ in solution. In contrast, the sizes of c-P(St-MPS) stabilized emulsion droplets are all smaller than those of t-P(St-MPS) because of the higher morphological asymmetry of the cap particles.

### 2.4. Applications of P(St-co-MPS)@Ag Particles and Kinetics of Catalytic 4-NP Reduction

**Figure 11:**
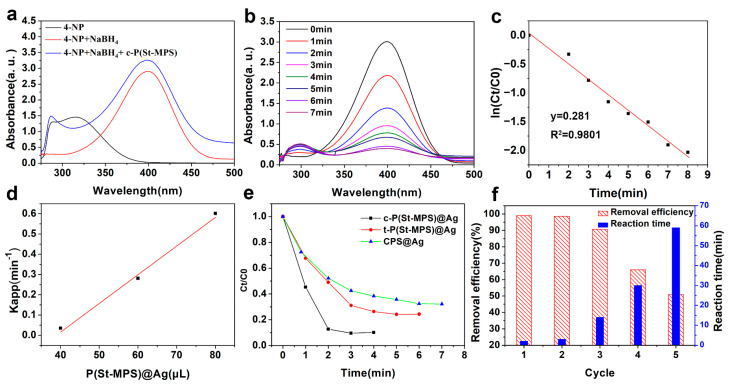
The catalytic performance of P(St-MPS)@Ag: (**a**) UV absorption peak of 4-NP, 4-NP+NaBH_4_; (**b**) UV–Vis spectra every one minute after the addition of P(St-MPS)@Ag; (**c**) catalytic kinetics; (**d**) The relationship between Kapp and concentration; (**e**) Degradability of 4-Np under different particles; (**f**) Five catalytic cycles of 4-NP degradation.

The catalytic performance of P(St-MPS)@Ag was evaluated when modeled on 4-NP organic pollutants. Freshly prepared NaBH_4_ was added to the 4-NP solution, which became bright yellow and alkaline, ionizing 4NP, and changing its UV absorption peak from 317 nm to 400 nm, as shown in Figure 11a. When the capsule particle solution was added to the 4NP+NaBH_4_ solution, the UV absorption values further increased due to the increase in the concentration of the solution, as the polystyrene-forming component of P(St-MPS) showed an absorption peak at 270 nm–380 nm. Although the blank cap particles can adsorb a small amount of 4-NP, they are unable to degrade it, as shown in Figure S8. When catalyst P(St-MPS)@Ag was added to the 4NP solution, as shown in Figure 11b, the absorption peak at 400 nm decreased with the reaction time, while another absorption peak appeared at 300 nm, indicating that 4NP was reduced to 4AP. Additionally, iso-absorption points were observed at 280 nm and 314 nm, indicating the absence of by-products.

(1) $$ln\frac{Ct}{C0}=ln\frac{At}{A0}=-K\mathrm{app}*t,$$

where *K*app is the apparent rate coefficients; and *Ct* and *C*0 are the concentrations of 4-NP at the time of *t* and the beginning of the reaction, respectively.

As shown in Figure 11c, t and ln Ct/C0 are linearly related, so the catalytic reaction conforms to the first-order kinetic equation. The linearity of t versus 1nCt/C0 was further assessed by adding different volumes of c-P(St-MPS)@Ag (Figure S8b) and the corresponding Kapp values were calculated (Figure 9d). The catalytic performance of 4-NP was compared with the catalytic performance of prepared cap-shaped particles, three-sided concave particles, and uniform spherical CPS particles loaded with Ag at the same MPS concentration. The catalytic rate of c-P(St-MPS)@Ag was much larger than that of c-P(St-MPS)@Ag and CPS@Ag at the same time (Figure 11e). The cap-shaped P(St-MPS)@Ag Janus nanoparticles are shown to maintain the catalytic activity of Ag while enhancing it through their morphological structure.

Recyclability is also an important parameter of the catalyst, and after the degradation process is complete, Janus P(St-MPS)@Ag can be recovered by simple centrifugation. Figure 11f demonstrates that c-P(St-MPS)@Ag exhibits some recoverability and recyclability. After three cycles, it retains about 90% of its original catalytic performance. However, in the last two cycles, there has been a notable increase in catalytic reaction time and a decrease in performance, attributed to the loss of centrifugally washed pellets.

## 3. Materials and Methods

### 3.1. Materials

Potassium persulfate (KPS), silver nitrate (AgNO_3_), 4-nitrophenol (4-NP), sodium borohydride (NaBH_4_), divinyl benzene (55%, 1000 ppm, TBC Stabilizer, DVB), and triethylamine were obtained from Aladdin (Shanghai, China). Methyl methacrylate (MMA), toluene, anhydrous ethanol, 4-Styrenesulfonic acid sodium salt hydrate (NaSS), sodium chloride (NaCl), hydrochloric acid (HCl, the mass fraction is approximately 37%), and caustic soda (NaOH)were provided Tianjin Komeo Chemical Reagent Co., Ltd. (Tianjin, China). Styrene (St, 99%) and acrylic acid (AA) were supplied by Tianjin Damao Chemical Reagent Factory (Tianjin, China). 3-methacryloxypropyltrimethoxysilane (MPS) was obtained from Macklin (Shanghai, China). Distilled water (H_2_O) was prepared in our lab.

### 3.2. Preparation of PS Seed Particles

Non-crosslinked monodispersed PS seed particles were synthesized by soap-free emulsion polymerization. Briefly, 100 mL of deionized water was poured into a 250 mL three-necked round bottom flask equipped with a stirring rod. Then, 10 mL of St was added after deoxygenation bubbled with N_2_ for 30 min at room temperature. The flask containing the mixture was heated to 70 °C, then 50 mL aqueous solution with 225.5 mg KPS (2.5 wt%) was added drop by drop within 30 min to initiate the polymerization, and the reaction was performed for 8 h with stirring at 600 rpm. Afterward, the PS particles were separated from the emulsion solution by centrifugation at 10,000 rpm and washed three times with distilled water. Finally, the PS particles are dispersed in a small amount of distilled water and stored for later use.

### 3.3. Synthesis of Anisotropic Hybrid Particles

#### 3.3.1. Synthesis of Cap-like P(St-MPS) Particles

The general procedure for preparing cap P(St-MPS) particles is 4.51 g of PS seed emulsion with a mass fraction of 10 wt% and 20 mL of deionized water was added to a 100 mL three-necked flask. After nitrogen bubbling for 30 min, at a 4:1 mass ratio of St-to-PS seed, 2 mL of St and 37 mg of MPS (2 wt% of St mass) were added and dissolved for 1 h at 25 °C with stirring at 500 rpm. After the temperature reached 65 °C, polymerization was initiated by adding an aqueous solution containing 45.1 mg of KPS (2.5 wt% of St mass), and the total volume of the mixed system was adjusted to 40 mL. After keeping the reaction for 8 h, the reaction mixture was cooled to room temperature. The asymmetric P(St-MPS) particles were separated from the emulsion by centrifugation at 10,000 rpm for 3 min. They were then rinsed three times with distilled water, dispersed in water, and set aside for later use.

#### 3.3.2. Synthesis of Spherical P(St-MPS) Particles

The amounts and proportions of PS seeds, St, MPS, and KPS are the same as in Section 3.3.1, except that the solvent environment is changed to the same volume of NaSS solution (0.1 wt%) or ethanol–water mixture (1:1 *v*/*v*) solution for the preparation of spherical P(St-MPS) polymer particles.

#### 3.3.3. Synthesis of Microphase-Separated PS@PMPS Core–Shell Particles

Microphase-separated PS@PMPS core–shell particles were synthesized by incorporating 37 mg of MPS as the sole reaction monomer while using 4.51 g of PS seed emulsion and maintaining the identical reaction conditions described in Section 3.3.1.

#### 3.3.4. Synthetic Hydrophilic Spherical Crosslinked Polystyrene (CPS) Particles

The preparation of CPS particles was carried out in the literature [29] with modifications, and the ratio of monomers was the same as in Section 3.3.1. Briefly, 20 mL of deionized water was added to a 100 mL three-necked flask, nitrogen bubbled for 30 min to remove oxygen, 37 mg of MPS was added and stirred for 30 min to hydrolyze it, 2 mL of St was added, stirred to raise the temperature to 65 °C, 20 mL of KPS aqueous solution was added (KPS was 2.5 wt% of the mass of St), reacted for 8 h, reduced to room temperature, centrifuged at 10,000 rpm for 3 min, and the separation was repeated 2 times and dispersed in water.

#### 3.3.5. Synthesis of Cap-like P(St-MPS-AA) Particles

Similar to the above procedure 3.3.1, 4.51 g of PS seed emulsion with a mass fraction of 10 wt% and 20 mL of deionized water was added to a 100 mL three-necked flask, and nitrogen bubbling for 30 min. Cap-like P(St-MPS-AA) particles were prepared by adding 1.9 mL of St, 0.1 mL of AA, 37 mg of MPS dissolved for 1 h at 25 °C, warmed to 65 °C, and then poured into an aqueous solution containing 45.1 mg of KPS to give a total mixture volume of 40 mL, and it was polymerized 8 h.

#### 3.3.6. Synthesis of PS@P(MMA-MPS) and PS@P(AA-MPS) Core–Shell Particles

Similar to the above procedure in Section 3.3.1, at the same monomer to PS seed mass ratio, MPS and KPS addition, with all other reaction conditions remaining unchanged, PS@P(MMA-MPS) and PS@P(AA-MPS) particles were prepared after replacing the solvated polymerization monomer St with the same mass of MMA and AA, respectively.

#### 3.3.7. Synthesis of PS@P(St-DVB)

Similar to the above procedure in Section 3.3.1, at the same monomer-to-PS seed mass ratio, and KPS addition, maintaining other conditions, the crosslinker MPS was replaced with the same mass of DVB, and PS@P(St-DVB) particles were obtained after polymerization.

### 3.4. Preparation of P(St-MPS)@Ag Composites Particles

The P(St-MPS) @Ag composite particles were synthesized using an in situ reduction method. Specifically, 0.3 g of P(St-MPS) particles were dispersed in 40 mL of distilled water in a 100 mL flask, and 5 mL of silver nitrate solution at a concentration of 0.01 g/L was added and stirred at room temperature for 30 min. Then, 1 mL of triethylamine was added to the mixture and the reaction was continued at 25 °C for 12 h. Once the reaction had finished, the solution was washed by centrifugation 3 times at 10,000 rpm in distilled water for 5 min, dried under vacuum at 60 °C, and then configured as a 1 mg/mL solution for spare use.

### 3.5. Study of Emulsion Stability

PS, CPS, and anisotropic P(St-MPS) particles were used as particulate emulsifiers to evaluate their emulsification performance on toluene/water-insoluble systems (toluene/water mass ratio of 1.6:1.4). Briefly, 0.01 g of particles were ultrasonically dispersed into 1.4 g of water, 1.6 g of toluene was added to the dispersion, and the particles were mixed by vortex mixer at high speed for 3 min at room temperature so that the particles came into full contact with the toluene/water. It was left to stand, and the height of the emulsified layer was observed and recorded at different times. The emulsion stability performance of the particles was evaluated using the emulsification value index (*EI*) [30]. The formula of *EI* is shown below.

(2) $$EI=\frac{H_{1}}{H}\times 100\%,$$

*H*_1_: Emulsion layer height.
*H*: Total height of the hybrid system.

### 3.6. Study of Catalytic Performance

The catalytic reduction of 4-NP by anisotropic P(St-MPS)@Ag composite particles was used as catalysts and was monitored using UV−vis spectroscopy. The following procedure was 0.1 mL of 4-NP (1 g/L),2.3 mL of DI water, and 0.1 mL of P(St-MPS)@Ag, which were successively added to the quartz dish. Moreover, 0.5 mL of freshly prepared NaBH4 in an ice-cold water solution (0.2 M) was added to initiate the catalytic reaction. The changes in the UV absorption peak were monitored every minute until the reaction was complete.

To assess the catalytic recyclability of P(St-MPS)@Ag, the concentrations of 4-NP andNaBH_4_ were maintained at a constant level, while the volume of them was increased by a factor of 5.1 mL of P(St-MPS)@Ag was introduced into the system, and made the total volume of the reaction system 15 mL. The degradation time required for the 4-NP+NaBH_4_ mixture to transition from a bright yellow to a colorless state was meticulously recorded. Upon completion of each reaction, the catalyst was subjected to washing via centrifugation to prepare for the subsequent cycle. The absorbance of the supernatant, obtained after separation, was determined using a UV-Vis spectrophotometer. The removal efficiency (R) was calculated using the Formula (3):

(3) $$R=\left(1-\frac{Ae}{A0}\right)\times 100\%$$

where *Ae* and *A*0 are the concentrations of 4-NP at the end and beginning of the reaction, respectively.

### 3.7. Characterization

The morphology and size distribution were observed by transmission electron microscopy (TEM, Thermo Fisher Scientific Talos F200X, Waltham, MA, USA), scanning electron microscopy (SEM, Carl Zeiss TM3000 Hitachi-Hightech, Krefeld, Germany, and FEI Quanta 450FEG, FEI Company, Hillsboro, OR, USA), and energy-dispersive X-ray (EDX). A Fourier-transform infrared spectroscope (FTIR, Bruke EQUINOX-55, Bruker Optics GmbH, Ettlingen, Germany) was utilized to investigate the chemical structures. Dynamic light scattering (DLS, BIC Nano Brook 90 plus PALS, Holtsville, NY, USA) analyzed the diameters of particles. The crystal structures were obtained by X-ray diffraction (XRD, Bruke D8 Quest, Bruker AXS GmbH, Karlsruhe, Germany), and the instrument was operated with a Cu Kα X-ray source at 40 kV and 100 mA. UV−vis absorption spectra were performed using a UV−vis spectrophotometer (UV-1900, Shanghai Helper International Trading Co., Shanghai, China). The thermal weight loss curves of the samples were analyzed using a thermogravimetric analyzer (TG, NETZSCH TG-209, Netzsch, Germany). After dispersing the toluene emulsion layer stabilized by cap particles with toluene, optical photographs (OLYMPUS IX73, Tokyo, Japan) were taken with an inverted fluorescence microscope at a magnification of 4 times. A No. 5 filter was used during the photography.

## 4. Conclusions

Anisotropic particles such as core–shell, bowl, cap, and three-sided concave were prepared by the interfacial polymerization of soap-free seed emulsion, and the key factors regulating the morphology of the particles were investigated. The results showed that the formation of asymmetric particles is crucially dependent on the partial wetting of hydrophilic MPS on the surface of hydrophobic seed droplets in the absence of soap. Additionally, kinetic factors such as the reactivity of MPS with St and the rapid initiation of polymerization from the aqueous phase by a high concentration of KPS causing a reaction rate difference between the inside and outside of the droplet also play an important role. The topology of P(St-MPS) can be controlled by fine-tuning MPS addition and St monomer. The cap particles are internal and external asymmetric Janus particles, which can be used as particle emulsifiers to stabilize p-toluene/water to form oil-in-water emulsions, with the excellent stabilization of toluene emulsion droplets even under harsh conditions such as excessive acidity, alkalinity, or high-salt. The -Si-OH on its surface effectively loads and disperses Ag nanoparticles with better catalytic efficiency for 4-NP based on the asymmetry of the cap particles, and the particles can be recovered after centrifugal separation. The results of this study provide new preparation strategies and design schemes for developing novel emulsifiers and carrier particles for use in emulsion stabilization and catalysis. Future work will focus on further investigating the preparation of anisotropic particles with varying compositions. Additionally, the exceptional emulsifying properties of the cap particles will be harnessed for more in-depth application studies, particularly in the realm of heterogeneous catalysis.

## Data Availability

Data are available on request from the authors.

## Supplementary Materials

The following supporting information can be downloaded at: https://www.mdpi.com/article/10.3390/molecules30010166/s1, Figure S1: Mean particle size and DLS particle size distribution of PS seeds; Figure S2: SEM (a), TEM (b,c), and EDS (d) tests of P(St-MPS)@Ag particles; Figure S3: The reaction and statistics on the change in the size of the samples during the reaction; Figure S4: The EDS maps of c-P(St-MPS) (a) and t-P(St-MPS) (b); Figure S5: The TG curves of the PS seed, c-P(St-MPS), and t-P(St-MPS); Figure S6: Photographs of PS, CPS, cP(St-MPS), and tP(St-MPS) stabilized toluene/water emulsions and trends in EI values over 30 days; Figure S7: Optical microscope photographs of three-sided concave particles stabilizing toluene emulsion droplets in different pH solutions; Figure S8: (a) Adsorption curve of c-P(St-MPS) on 4-NP+NaBH_4_, (b) The relationship between ln (Ct/C0) and the reaction time.

## References

[1] He X., Liang C., Liu Q., Xu Z. (2019). Magnetically responsive Janus nanoparticles synthesized using cellulosic materials for enhanced phase separation in oily wastewaters and water-in-crude oil emulsions. Chem. Eng. J..

[2] Hou Y., Li Y., Wang L., Chen D., Bao M., Wang Z. (2019). Amphiphilic Janus particles for efficient dispersion of oil contaminants in seawater. J. Colloid Interface Sci..

[3] Bao Y., Zhang Y., Ma J. (2020). Reactive amphiphilic hollow SiO_2_ Janus nanoparticles for durable superhydrophobic coating. Nanoscale.

[4] Hao Y., Bai S., Yu L., Sun Y. (2022). Magnetically Driven Muco-Inert Janus Nanovehicles for Enhanced Mucus Penetration and Cellular Uptake. Molecules.

[5] Wang S., Liu H., Wu Q., Shu Y., Wang Q., Zhang G., Xu L., Liang F. (2024). Catalytic Janus Nanoparticle-Based Recyclable Emulsifiers for Collaborative Treatment of Water-Soluble and Water-Insoluble Organic Pollutants. ACS Appl. Nano Mater..

[6] Chen C., Zhang L., Wang N., Sun D., Yang Z. (2023). Janus Composite Particles and Interfacial Catalysis Thereby. Macromol. Rapid Commun..

[7] Walther A., Muller A.H. (2013). Janus particles: Synthesis, self-assembly, physical properties, and applications. Chem. Rev..

[8] Yang L., Xu J., Wang J., Lang F., Liu B., Yang Z. (2020). Responsive Single-Chain/Colloid Composite Janus Nanoparticle. Macromolecules.

[9] Agrawal G., Agrawal R. (2019). Janus Nanoparticles: Recent Advances in Their Interfacial and Biomedical Applications. ACS Appl. Nano Mater..

[10] Duan Y., Zhao X., Sun M., Hao H. (2021). Research Advances in the Synthesis, Application, Assembly and Calculation of Janus Materials. Ind. Eng. Chem. Res..

[11] Pham B.T.T., Such C.H., Hawkett B.S. (2015). Synthesis of polymeric Janus nanoparticles and their application in surfactant-free emulsion polymerizations. Polym. Chem..

[12] Sfika V., Tsitsilianis C. (2004). pH Responsive Heteroarm Starlike Micelles from Double Hydrophilic ABC Terpolymer with Ampholitic A and C Blocks. Macromolecules.

[13] Kim J.W., Larsen R.J., Weitzd A. (2007). Uniform Nonspherical Colloidal Particles with Tunable Shapes. Adv. Mater..

[14] Kim J.W., Larsen R.J., Weitzd A. (2006). Synthesis of nonspherical colloidal particles with anisotropic properties. J. Am. Chem. Soc..

[15] Mock E.B., Bruyn H.D., Hawkett B.S., Gilbert R.G., Zukoski C.F. (2006). Synthesis of Anisotropic Nanoparticles by Seeded Emulsion Polymerization. Langmuir.

[16] Wang L., Pan M., Song S., Zhu L., Yuan J., Liu G. (2016). Intriguing Morphology Evolution from Noncrosslinked Poly(*tert*-butyl acrylate) Seeds with Polar Functional Groups in Soap-Free Emulsion Polymerization of Styrene. Langmuir.

[17] Song Y., Wan X., Wang S. (2023). Heterostructured Microparticles: From Emulsion Interfacial Polymerization to Separation Applications. Acc. Mater. Res..

[18] Cheng Z., Luo F., Zhang Z., Ma Y. (2013). Syntheses and applications of concave and convex colloids with precisely controlled shapes. Soft Matter..

[19] Song Y., Zhou J., Fan J.-B., Zhai W., Meng J., Wang S. (2018). Hydrophilic/Oleophilic Magnetic Janus Particles for the Rapid and Efficient Oil-Water Separation. Adv. Funct. Mater..

[20] Zhao K., Hu J., Ma Y., Wu T., Gao Y., Du F. (2019). Topology-Regulated Pesticide Retention on Plant Leaves through Concave Janus Carriers. ACS Sustain. Chem. Eng..

[21] Fan J.-B., Liu H., Song Y., Luo Z., Lu Z., Wang S. (2018). Janus Particles Synthesis by Emulsion Interfacial Polymerization: Polystyrene as Seed or Beyond?. Macromolecules.

[22] Zhai W., Song Y., Gao Z., Fan J.-B., Wang S. (2019). Precise Synthesis of Polymer Particles Spanning from Anisotropic Janus Particles to Heterogeneous Nanoporous Particles. Macromolecules.

[23] Sun H., Lin S., Ng F.T.T., Mitra S.K., Pan Q. (2021). Synthesis of Shape-Controllable Anisotropic Microparticles and “Walnut-like” Microparticles via Emulsion Interfacial Polymerization. Langmuir.

[24] Xie L., Liu T., He Y., Zeng J., Zhang W., Liang Q., Huang Z., Tang J., Liang K., Jiang L. (2022). Kinetics-Regulated Interfacial Selective Superassembly of Asymmetric Smart Nanovehicles with Tailored Topological Hollow Architectures. Angew. Chem. Int. Ed..

[25] Park J.J., Kim Y., Lee C., Kim D., Choi W., Kwon H., Kim J.H., Hwang K.S., Lee J.Y. (2021). Morphological Analysis of PSMA/PEI Core–Shell Nanoparticles Synthesized by Soap-Free Emulsion Polymerization. Nanomaterials.

[26] Elham D., Mehdi S.-K., Hossein R.-M. (2019). Fabricating cauliflower-like and dumbbell-like Janus particles: Loading and simultaneous release of DOX and ibuprofen. Colloids Surf. B Biointerfaces.

[27] Morgenthaler E.C., Ribbe A.E., Bradley L.C., Emrick T. (2025). Alkyne-rich patchy polymer colloids prepared by surfactant-free emulsion polymerization. J. Colloid Interface Sci..

[28] Sun Y., Zhang H., Han W., Huang H., Qi D. (2019). Controllable synthesis of monodisperse nonspherical colloidal particles with cavity structures. J. Polym. Sci..

[29] Nagao D., Hashimoto M., Hayasaka K., Konno M. (2008). Synthesis of Anisotropic Polymer Particles with Soap-Free Emulsion Polymerization in the Presence of a Reactive Silane Coupling Agent. Macromol. Rapid Commun..

[30] Tan J.S.J., Wong S.L.Y., Chen Z. (2020). Preparation of Janus Titanium Dioxide Particles via Ultraviolet Irradiation of Pickering Emulsions. Adv. Mater. Interfaces.

