# Supplementary material for: Anisotropic Microparticles with a Controllable Structure via Soap-Free Seeded Emulsion Polymerization
Source: Molecules. 2025 Jan 3;30(1):166. doi: 10.3390/molecules30010166 (PMC11721275; doi:10.3390/molecules30010166)
Supplement: Supplementary file 1 [file molecules-30-00166-s001.zip › molecules-3392220-supplementary.pdf]

Supplementary Materials

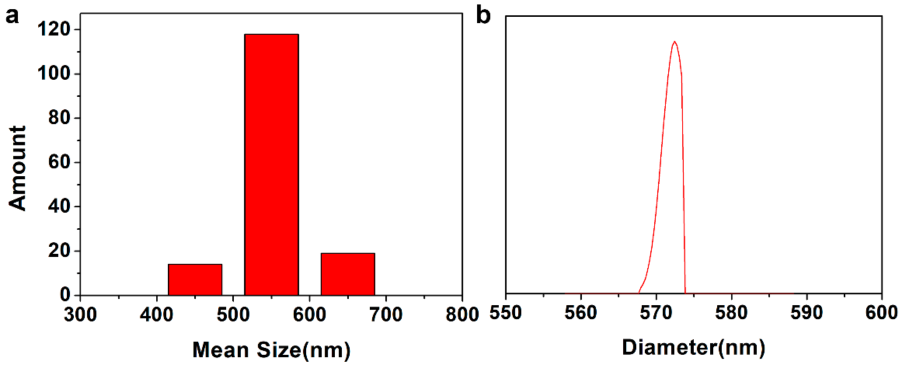

Figure S1. Mean particle size and DLS particle size distribution of PS seeds (**a**,**b**).

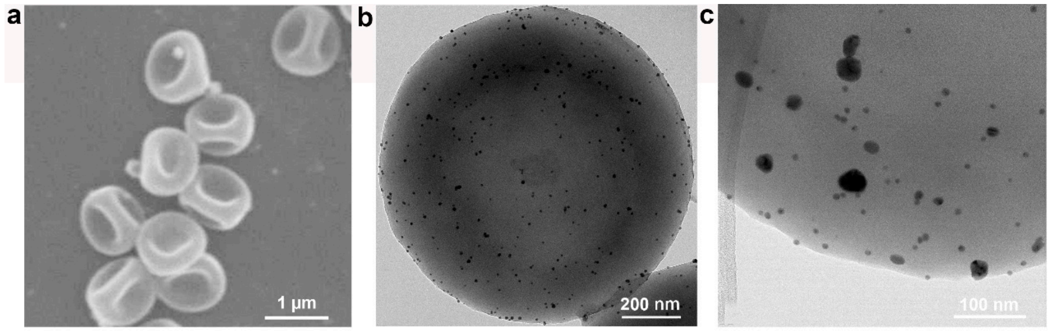

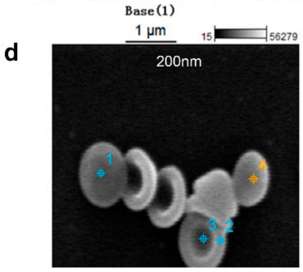

Wt%	C	O	Si	Ag
pt1	17.26±10.04	43.66±2.52	38.57±1.28	0.51±1.10
pt2	0.00±0.00	50.91±2.36	48.46±1.64	0.63±1.68
pt3	7.94±12.14	49.90±2.45	42.17±1.32	0.00±0.00
pt4	8.05±11.02	49.41±1.61	41.77±1.28	0.77±1.00

Figure S2. SEM) (**a**), TEM (**b**,**c**), and EDS (**d**) tests of P(St-MPS)@Ag particles.

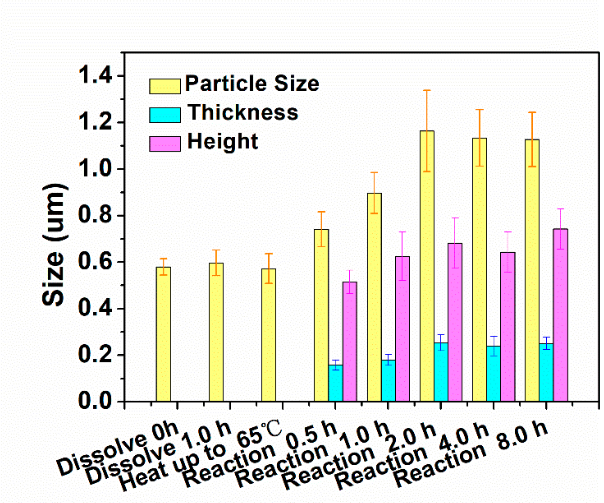

Figure S3. The reaction and statistics on the change in the size of the samples during the reaction.

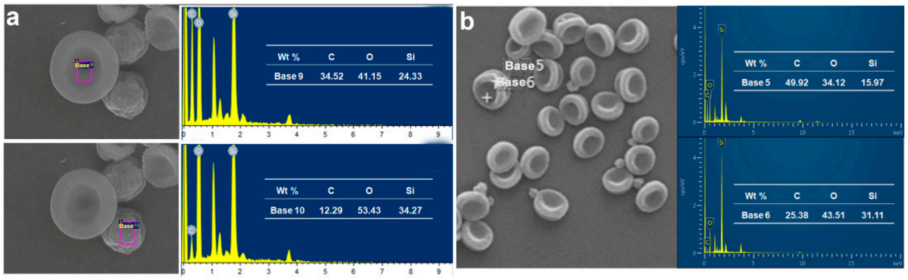

Figure S4. The EDS maps of c- P(St-MPS) (**a**) and t- P(St-MPS) (**b**).

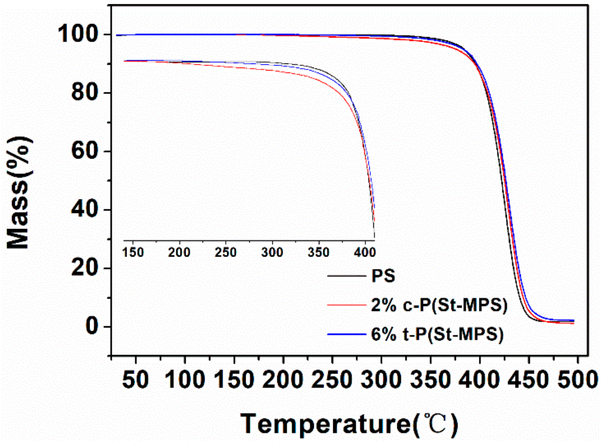

Figure S5. The TG curves of the PS seed,c-P(St-MPS) and t-P(St-MPS).

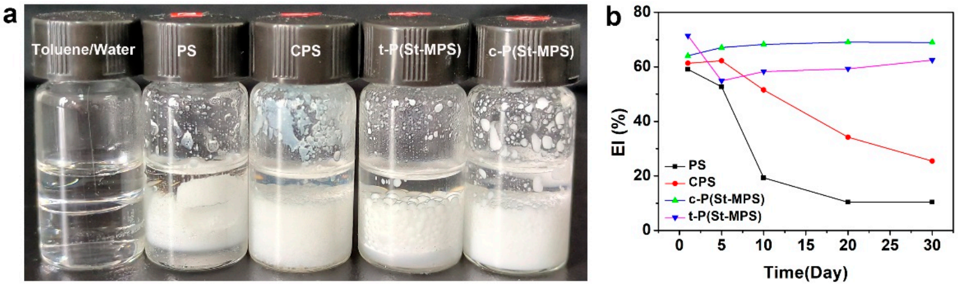

Figure S6. Photographs of PS, CPS, c-P(St-MPS), and t-P(St-MPS) stabilized toluene/water emulsions and trends in EI values over 30 days (**a**,**b**).

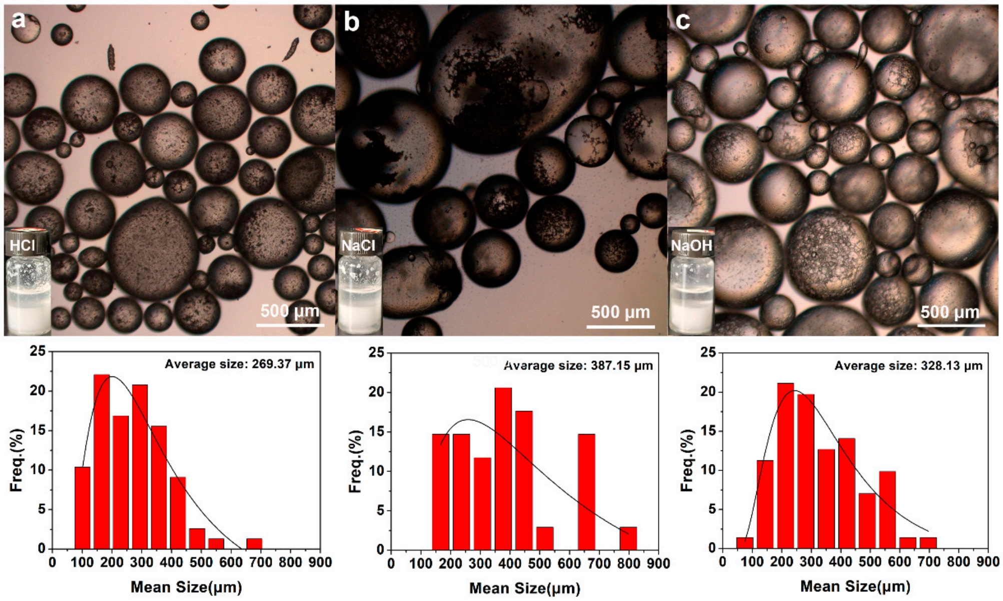

Figure S7. Optical microscope photographs of three-sided concave particles stabilizing toluene emulsion droplets in different pH solutions (**a**–**c**).

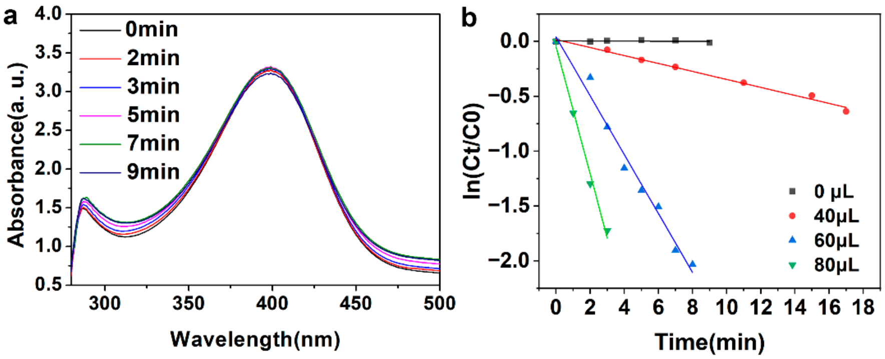

Figure S8. (**a**) Adsorption curve of c-P(St-MPS) on 4-NP+$NaBH_4$; (**b**)The relationship between ln (Ct/C0) and the reaction time.
